# Isolation and Identification of Cellulolytic Bacteria from the Gut of *Holotrichia parallela* Larvae (Coleoptera: Scarabaeidae)

**DOI:** 10.3390/ijms13032563

**Published:** 2012-02-23

**Authors:** Shengwei Huang, Ping Sheng, Hongyu Zhang

**Affiliations:** 1 State Key Laboratory of Agricultural Microbiology, Huazhong Agricultural University, Wuhan 430070, China; E-Mails: swhuang2006@yahoo.com.cn (S.H.); shengping_1014@163.com (P.S.); 2 Institute of Urban and Horticultural Pests, College of Plant Science and Technology, Huazhong Agricultural University, Wuhan 430070, China; 3 Hubei Insect Resources Utilization and Sustainable Pest Management Key Laboratory, College of Plant Science and Technology, Huazhong Agricultural University, Wuhan 430070, China

**Keywords:** 16S rDNA, ARDRA, gut microbiology, cellulose, biochemical and physiological tests, white grub

## Abstract

In this study, 207 strains of aerobic and facultatively anaerobic cellulolytic bacteria were isolated from the gut of *Holotrichia parallela* larvae. These bacterial isolates were assigned to 21 genotypes by amplified ribosomal DNA restriction analysis (ARDRA). A partial 16S rDNA sequence analysis and standard biochemical and physiological tests were used for the assignment of the 21 representative isolates. Our results show that the cellulolytic bacterial community is dominated by the *Proteobacteria* (70.05%), followed by the *Actinobacteria* (24.15%), the *Firmicutes* (4.35%), and the *Bacteroidetes* (1.45%). At the genus level, Gram-negative bacteria including *Pseudomonas*, *Ochrobactrum*, *Rhizobium*, *Cellulosimicrobium*, and *Microbacterium* were the predominant groups, but members of *Bacillus*, *Dyadobacter*, *Siphonobacter*, *Paracoccus*, *Kaistia*, *Devosia*, *Labrys*, *Ensifer*, *Variovorax*, *Shinella*, *Citrobacter*, and *Stenotrophomonas* were also found. Furthermore, our results suggest that a significant amount of bacterial diversity exists among the cellulolytic bacteria, and that *Siphonobacter aquaeclarae*, *Cellulosimicrobium funkei*, *Paracoccus sulfuroxidans*, *Ochrobactrum cytisi*, *Ochrobactrum haematophilum*, *Kaistia adipata*, *Devosia riboflavina*, *Labrys neptuniae*, *Ensifer adhaerens*, *Shinella zoogloeoides*, *Citrobacter freundii*, and *Pseudomonas nitroreducens* are reported to be cellulolytic for the first time in this study. Our results indicate that the scarab gut is an attractive source for the study of novel cellulolytic microorganisms and enzymes useful for cellulose degradation.

## 1. Introduction

Interest in bioenergy has been sharply increasing in recent years due to the necessity of sustainable economies and clean environments [1–3]. Cellulose and hemicellulose are the most abundant biomasses on earth, and therefore have the greatest potential to resolve both the energetic and environmental demands of bioenergy [4,5]. The production of ethanol and other biofuels from lignocellulosic biomass has recently received tremendous attention both in industry and in academic communities worldwide, and great progress has been made in the production and use of biofuels [1,3,6,7]. Lignocellulosic raw materials can be converted to ethanol by hydrolysis and subsequent fermentation [8]. In the hydrolysis step, acid-reliant hydrolytic processes have been used for many decades, but have also been blamed for negative effects on the environment, including the formation of large quantities of calcium sulfate that require disposal [3,7,8]. Thermochemical processes are another significant method of bioethanol production [9,10], but developing a cost-effective, all-thermochemical process has been difficult [11,12]. Consequently, enzymatic hydrolysis is a more environmentally sound approach [8], and the costs of this approach can be reduced with recent breakthroughs in molecular genetics, enzyme engineering and metabolic engineering, which has drawn greater attention from researchers [3,12–14]. In the fermentation step, the conversion of cellulose, the recovery efficiency and the cost depends strongly on the fermentation efficiency of the microorganisms and enzymes [15,16]. Currently, the mainstream process of bioethanol production makes use of the basic yeast *Saccharomyces cerevisiae* due to its unique advantages (e.g., genetically tractable, superior conversion yields of ethanol from glucose, high alcohol tolerance) [17,18]. However, the rising concentration of sugars and aromatic components in the industrial conversion of lignocellulose to ethanol as well as other adverse conditions can cause harm to the *S. cerevisiae* and restrict its applications [18]. Therefore, isolating ideal biofuel-producing microorganism with ability to degrade different lignocellulosic materials, resist different inhibitors and biosynthesize specific biofuels with high yield has an important role in developing biofuel production systems [3,18]. Some insects, such as termites, wood-feeding roaches, beetles, and leaf-cutting ants, can use lignocellulosic substrates as their main food source and are highly efficient at degrading cellulose to glucose as an energy source [1]. There have been numerous reports on the cellulolytic activity of these insects, which include *Reticulitermes flavipes* [19], *Anoplophora glabripennis* [20], *Tenebrio molitor* [21], and *Pachnoda marginata* [22]. The gut systems of these insects are diverse and highly adapted and are considered to be highly efficient natural bioreactors [1,4,23]. Furthermore, the intestinal microorganisms of these lignocellulose-degrading insects are considered to be essential for cellulose digestion. Gut microbiota symbiotically associated with lower termites are responsible for the decomposition of various kinds of organic matter and for biomass recycling [24–26]. Additionally, a number of protozoa and bacteria with cellulolytic activity have been isolated from *Zootermopsis angusticollis* [25], silver cricket *Lepisma sp*. [27], *Tipula abdominalis* [28], *Saperda vestita* [29], *Dendroctonus frontalis* [29], and *Pachnoda marginata* [30]. These studies suggest that lignocellulose-degrading insects are an attractive potential source of novel cellulolytic microorganisms and enzymes and suggest that these enzymes may be useful in biofuel production [28,31].

The family Scarabaeidae, as currently defined, comprises over 30,000 species of beetles, which are almost exclusively herbivorous or saprophagous [31]. Many scarab larvae live in the soil and feed on roots or other organic matter [32]. The hindgut of scarab larvae (also referred to as the fermentation sac) is enlarged and houses dense microbial communities [33,34]. Previous studies have shown that 25–65% of the ingested pure cellulose or neutral detergent fibers in their diet are degraded by scarab larvae and that the intestinal bacteria in the hindgut of these larvae are responsible for cellulose degradation [22,35]. Furthermore, several cellulolytic bacterial species have been successfully isolated from the gut contents of some scarab species [30,36]. These studies demonstrated that the hindgut of scarab larvae represent an ideal prospecting resource for identifying microorganisms and enzymes that can be used for biofuel production and to improve biofuel production technology [31].

The root-feeding larvae of *Holotrichia parallela* live in the soil in China and feed on a variety of plants, resulting in significant economic damage [37]. In this study, we isolated and identified cellulose-degrading bacteria from the gut of *H. parallela* to obtain a more precise estimation of their occurrence in scarabs, and we specifically evaluated the nutritional contributions of gut microbiota to scarabs, and also assessed their potential to future biofuel production.

## 2. Results and Discussion

### 2.1. Isolation of Cellulolytic Bacteria

Bacteria with CMCase activity were abundant (1.14 ± 0.13 × 10^8^ colony forming units (CFU)/gut) in the hindgut of *H. parallela*. However, bacteria with CMCase activity were seldom found in the midgut (only 20 ± 1.45 CFU/gut). These results are similar to those from another scarab beetle, *P. marginata* [30]. Cazemier *et al.* [30] observed that a large number of bacteria with CMCase and xylanase activities were present in the hindgut of *P. marginata* (2.5 ± 1.1 × 10^8^ CFU (mL_gut_) ^−1^), but that these bacteria were not detected in the midgut. Studies of the gut microbiota of other scarab beetles showed that the hindgut microbiota was dominated by groups of fermentative bacteria such as *Clostridiales*, *Actinobacteria*, and *Cytophaga*-*Flavobacterium*-*Bacteroides*, which contain a wide range of species able to ferment cellulose, hemicellulose, pectin and other polysaccharides [34,38–40]. These results indicate that the bacteria in the scarab hindgut play an important role in the degradation of roots and other organic matter consumed by scarab larvae, as suggested by Cazemier *et al*. [30] and Huang *et al*. [31]. As bacteria with cellulolytic activity appear to be absent in the midgut, it seems likely that the midgut of scarabs serves a predigestive function for lignocellulose rather than for the microbial degradation of cellulose and hemicellulose [30].

**Figure 1:**
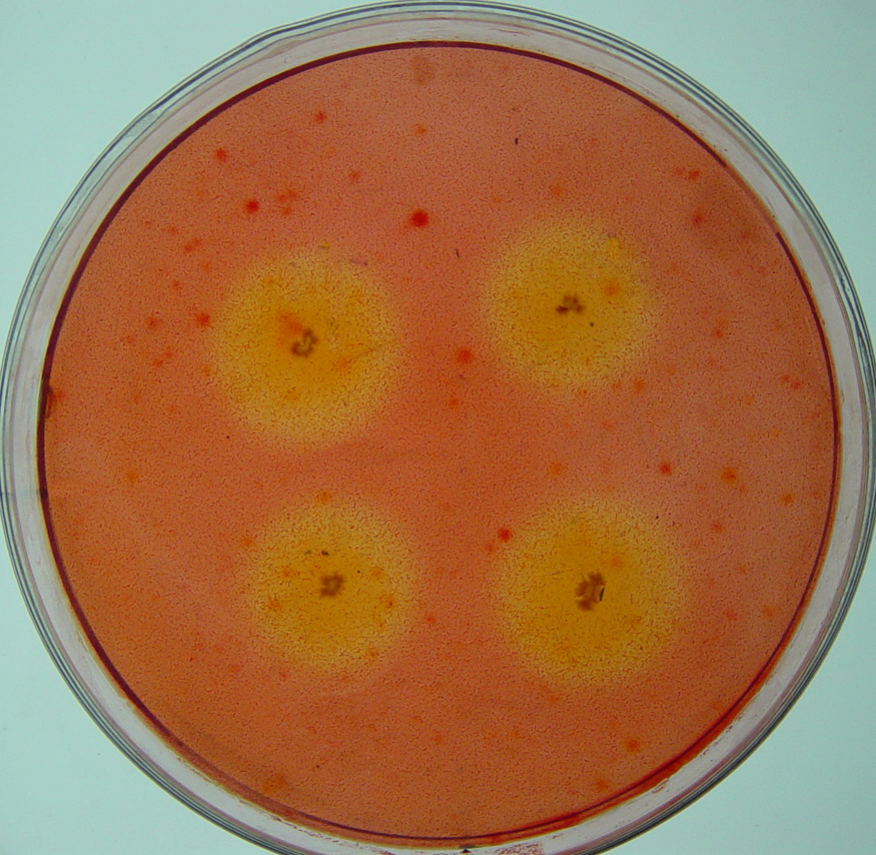
Screening of cellulolytic bacteria by covering the petri dishes with congo red dye. A zone of clearance surrounding a colony is indicative of carboxymethylcellulose (CMC) hydrolysis by secreted CMCase.

**Figure 2:**
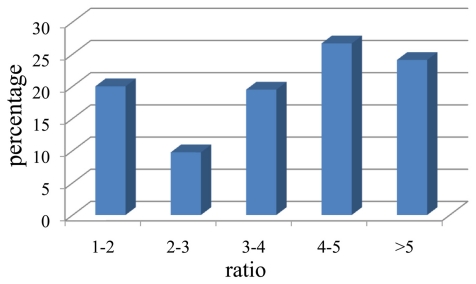
The ratio of the diameter of the zone of clearance to the diameter of the colony and the percentage of the bacterial isolates in each range of ratios.

A total of 207 isolates with CMCase activity were obtained from the gut contents of *H. parallela* either by plating on CMC medium or by enrichment on filter paper. Among these cellulolytic bacteria, 81 isolates were obtained using the filter paper inoculation method, and 126 isolates were obtained from direct plating. These isolates produced variable zones of CMC clearance (Figure 1). Based on the calculation of the ratio of the diameter (mm) of the zone of clearance to the diameter of the colony, it was determined that these bacterial isolates demonstrated large differences in their ability to degrade CMC (Figure 2). This ratio ranged from 1.1 to 9.0 among all the isolates, with 24.1% of the isolates showing high CMC-degrading activity (ratio > 5), demonstrating that multiple bacterial isolates from the scarab gut possess the ability to produce CMCase (Figure 2).

### 2.2. Assignment and Identification of Cellulolytic Bacteria

The 207 cellulolytic bacterial isolates obtained in this study were grouped into 21 clusters or genotypic groups (Table 1). Each group displayed a specific ARDRA banding pattern, and the number of isolates belonging to each group was different (Table 1). A total of 21 isolates were chosen to represent each ARDRA group, and these isolates were investigated both by 16S rDNA sequencing and by physical and biochemical characterization (Table 2). Overall, the 16S rDNA sequences from the 21 isolates showed a high degree of similarity (99–100%) to a number of annotated sequences found in the databases (data not shown), and their identification was in agreement with the biochemical and physiological tests. The 21 isolates clustered into four phyla (*Proteobacteria*, *Actinobacteria*, *Bacteroidetes*, and *Firmicutes*), and represented 17 different genera (Table 1). The cellulolytic bacterial community was represented by members of the phylum *Proteobacteria* (67.13%), followed by *Actinobacteria* (23.15%), *Firmicutes* (4.35%), and *Bacteroidetes* (1.45%). At genus level, *Pseudomonas* (31.4%), *Cellulosimicrobium* (13.53%), *Ochrobactrum* (12.08%), *Rhizobium* (11.59%), and *Microbacterium* (10.63%) were the dominant genera identified, with 65, 28, 25, 24 and 22 isolates, respectively, while *Siphonobacter* (group 3), *Devosia* (group 14), *Variovorax* (group17), *Shinella* (group 18) each consisted of a single bacteria isolate. Furthermore, the ARDRA grouping results also revealed that bacterial isolates belonging to *Bacillus licheniformis*, *Microbacterium oxydans*, *Microbacterium binotii*, *Microbacterium aurum.*, *Cellulosimicrobium funkei*, *Ochrobactrum cytisi*, *Rhizobium radiobacter*, *Labrys neptuniae*, *Pseudomonas nitroreducens*, *Stenotrophomonas maltophilia* can obtained both by the direct plating method and by the filter papers enrichment method. The fact that these bacterial isolates can be obtained using both methods demonstrates that bacteria with cellulolytic ability are commonly present in the hindgut of *H. parallela*.

Our results showed that *Pseudomonas* was the most dominant group in the cellulolytic bacterial community in the gut of soil-dwelling scarab larvae. The dominance of *Pseudomonas* in the present study is similar to the results of previous studies on cellulolytic bacteria present on native Chaco soil, which showed that the *Pseudomonas* was the only genus that exists stably in three samples (native forest soil, CMC- and filter paper-enriched samples) [41]. Bacteria of the genus *Pseudomonas* can be found in many different environments including soil, water, plant and animal tissue, and these bacteria have the ability to metabolize a variety of diverse nutrients [42]. Many *Pseudomonas* species are opportunistic pathogens that infect humans, animals, and plants [43–45], but other *Pseudomonas* species also have been reported to degrade cellulose [46–48]. There have been no reports, however, describing the cellulolytic activity of *P. nitroreducens*, which we observed in this study.

The cellulolytic activity of some of the bacteria found in this study has been reported previously. *B. licheniformis* is characterized by strong xylanase activity, and also possesses CMCase, mannanase, and pectinase activities [49]. Though *Dyadobacter fermentans* NS114^T^ does not hydrolyze cellulose or starch [50], whole genome sequencing of *D. fermentans* DSM 18053 has revealed several genes encoding for 1,4-β-cellobiosidase, β-glucosidase, and *endo*-1,4-β-xylanase enzymes [51]. The *Microbacterium* genus contains many species with cellulolytic or xylanolytic activities. A cellulolytic bacterium that showed 99% 16S rDNA sequence similarity to *M. oxydans* has been found to produce an array of cellulolytic-xylanolytic enzymes (filter paper cellulase, β-glucosidase, xylanase, and β-xylosidase) [52]. *M. binotii* have also been reported to produce an enzyme with β-glucosidase activity [53]. *Rhizobium* species are known to produce cellulolytic and pectinolytic enzymes that can break the glycosidic bonds present in the plant cell wall, and these enzymes are essential for the primary symbiotic infection of legume host roots [54–56]. However, little attention has been paid to their potential ability to degrade organic compounds during their growth as free-living saprophytes [41,57]. An analysis of the genome sequence of *R. radiobacter* (formerly *Agrobacterium tumefaciens*) has identified several genes encoding pectinase, ligninase, and xylanase as well as genes encoding regulators of pectinase and cellulase production [58]. *Variovorax paradoxus* is a microorganism of special interest due to its diverse metabolic capabilities. Whole genome sequencing of *V. paradoxus* revealed a single gene encoding β-glucosidase, but genes involved in the production of pectinases and other cellulases remained unidentified [59]. The *Stenotrophomonas* genus contains species ranging from common soil organisms (*Stenotrophomonas nitritireducens*) to opportunistic human pathogens (*S. maltophilia*) [41]; one *S. maltophilia* strain from the mesophilic microbial community BYND-8 has also been reported to be cellulolytic [60].

In addition to those bacterial isolates for which cellulolytic activity has been well described, our results demonstrate cellulolytic activity for several bacterial strains that have not been previously reported to be cellulolytic. To the best of our knowledge, this is the first report describing *Siphonobacter aquaeclarae*, *C. funkei*, *Paracoccus sulfuroxidans*, *O. cytisi*, *Ochrobactrum haematophilum*, *Kaistia adipata*, *Devosia riboflavina*, *L. neptuniae*, *Ensifer adhaerens*, *Shinella zoogloeoides*, *Citrobacter freundii*, and *P. nitroreducens* as being cellulolytic, with some isolates displaying high cellulolytic activity. In the case of *S. aquaeclarae*, the ratio of the CMC clearance zone to the colony diameter was greater than 7, and for *C. funkei*, the ratio ranged from 3.3 to 5.3, indicating robust CMC-ase production. These cellulolytic bacterial isolates demonstrate great potential for the study of novel enzymes in cellulose degradation and for improving the bioconversion of lignocellulosic biomass.

## 3. Experimental Section

### 3.1. Insect and Dissection

Third-instar larvae of *H. parallela* were collected from a peanut field and were maintained individually in containers with sterile soil. All the larvae were fed with peanuts surface sterilized with 70% ethanol, and the diets were replaced every 3 days. After 3 weeks, 9 healthy larvae were surface sterilized with 70% ethanol to remove contamination, washed twice in sterile distilled water, and allowed to air dry for 1 min. The preparation of the intestinal tract (mid- and hindgut) was performed as described previously by Zhang and Jackson [34].

### 3.2. Media

Medium I and Medium II were prepared as described by Cazemier, *et al*. [30], with some modifications, as follows:

Medium I: peptone, 5 g/L; yeast extract, 0.1 g/L; K_2_HPO_4_, 1 g/L; MgSO_4_·7H_2_O, 0.2 g/L; carboxymethylcellulose (CMC), 10 g/L (sodium salt, low viscosity, Sigma); Na_2_CO_3_, 10 g/L (sterilized separately); pH 10.3.

Medium II: K_2_HPO_4_, 1.9 g/L; KH_2_PO_4_, 0.94 g/L; KCl, 1.6 g/L; NaCl, 1.43 g/L; NH_4_Cl, 0.15 g/L; MgSO_4_·7H_2_O, 0.037 g/L; CaCl_2_·2H_2_O, 0.017 g/L; yeast extract, 0.1 g/L; CMC, 10 g/L; pH 7.2.

Medium III was prepared as described by Wenzel *et al*. [25], with the following modifications: yeast extract, 0.04 g/L; malt extract, 0.1 g/L; CaCO_3_, 0.5 g/L; filter paper strips, 5 g/L (Whatman Filter Paper No.1); pH 10.3.

The media were sterilized (121 °C, 20 min) and solidified with agar (17 g/L) when necessary.

### 3.3. Counting and Isolation of Cellulolytic Bacteria

For viable counts, an individual gut was homogenized and suspended in 10 mL of medium I and serially diluted ten-fold (to 10^−9^). From each dilution, 100 μL was spread on plates with solid medium I (midgut) or medium II (hindgut). A triplicate series of dilutions from the midguts and hindguts of three different larvae were incubated at 28 °C. Colonies were counted following 4 weeks of incubation. Only the colonies that were encircled by a clear zone after staining with a solution of congo red (1 mg/mL) were counted.

To isolate cellulolytic bacteria, the midgut or hindgut sections from six individual larvae were pooled and homogenized. Serial dilutions and plating were performed as described above, and the plates were incubated aerobically at 28 °C for up to 4 weeks. In addition to directly plating the gut samples on solid media, 0.5 mL of the homogenized midgut or hindgut suspension was inoculated into 100 mL of medium III and incubated at 28 °C. After 3 weeks of enrichment, 100 μL of the growing cultures were cultivated on solid medium I (midgut) or medium II (hindgut). Bacteria from single colonies were repeatedly grown on solid agar plates until a pure culture was obtained.

### 3.4. CMCase Activity Assay

CMC degradation by the isolates was tested on solid medium II by covering the Petri dishes with congo red dye, as described by Teather and Wood [61]. Carboxymethylcellulose degradation was indicated by a clear zone around the colonies. Enzyme activity was indexed as the diameter of the colony plus the surrounding clear zone divided by the diameter of the colony [29]. Three measurements were taken from each isolate, and only the isolates that produced a clear zone around the colony were chosen for further study.

### 3.5. DNA Extraction and PCR Amplification of 16S rDNA

Bacterial isolates were grown in LB medium (Tryptone, 10 g/L; yeast extract, 5 g/L; NaCl, 10 g/L; pH 7.0) at 30 °C for 48 h. The cultures were centrifuged at 10,000× g for 1 min, and the supernatant was removed. DNA extraction was performed using a Cell/Tissue Genomic DNA Extraction Kit (BioTeke Corporation, Beijing, China) according to the manufacturer’s instructions, and the genomic DNA was stored at −80 °C until further analysis. Bacterial universal primers 27F (5′-AGAGTTT GATCMTGGCTCAG-3′) and 1492R (5′-TACGGYTACCTTGTTACGACTT-3′) were used to amplify the 16S rDNA from genomic DNA [62]. Polymerase chain reaction (PCR) was performed in a thermocycler (MyCycler, Bio-Rad, USA). Each reaction mixture (50 μL) contained 5 μL of 10× reaction buffer without MgCl_2_, 1.5 mM MgCl_2_, 0.2 μM of each primer, 0.2 mM of each dNTP, 2.5 U of Taq DNA polymerase (TaKaRa Biotechnology (Dalian) Co., Ltd., China), and 25 ng of template DNA. The amplification was performed as follows: initial denaturation for 5 min at 94 °C, 35 cycles each of denaturation for 30 s at 94 °C, annealing for 30 s at 55 °C, and primer extension for 1.5 min at 72 °C, and a final extension for 10 min at 72 °C. The PCR products were checked by gel electrophoresis in 1.2% (w/v) agarose gels stained with ethidium bromide (10 mg/mL) and cleaned using an EasyPure Quick Gel Extraction Kit (Transgen Biotech, China) according to the manufacturer’s instructions.

### 3.6. Genotyping of Bacterial Isolates by ARDRA

Amplified ribosomal DNA restriction analysis (ARDRA) was performed on the PCR-amplified 16S rDNA products from each of the isolates using three specific restriction enzymes: *Hha*I, *Afa*I, and *Msp*I (TaKaRa Biotechnology (Dalian) Co., Ltd., China). Five microliters of each PCR product was digested for 2 h at 37 °C with 1.5 U of each restriction endonuclease. Aliquots (5 μL) of each digested product were analyzed by gel electrophoresis in an 8% nondenaturing acrylamide gel (acrylamide: *N*,*N′*-Methylenebisacrylamide, 29:1) [63] and by silver nitrate staining, as described previously [64]. Fragment sizes were estimated using a low range, 50 bp DNA ladder (Dongsheng Biotech Co., Ltd., China), and a final grouping of isolates was performed by a visual comparison of the restriction patterns. For each distinct ARDRA group, one bacterial isolate was selected for sequencing and standard physical and biochemical characterization.

### 3.7. 16S rDNA Sequencing Analysis

Nearly full-length bacterial 16S rDNA fragments were amplified by PCR from each representative isolate using the universal primers 27F and 1492R, as described above. The PCR products were cleaned and cloned using the *pE*ASY-*T1* cloning kit (Transgen Biotech, China) with blue-white screening. The clones containing inserts of the correct size were sequenced, and the sequences were aligned against those found in the NCBI database [65], in the RDP II database [66], and on the EzTaxon server [67] using the BLAST (Basic Local Alignment and Search Tool) algorithm [68]. All the sequences have been submitted to the GenBank database under the accession numbers JQ291585-JQ291605.

### 3.8. Identification of Cellulolytic Isolates

For each ARDRA group, one representative isolate was identified based on standard physical and biochemical tests [69], including motility, Gram staining, the methyl red (MR) test, the Voges-Proskauer (VP) test, the activities of catalase, oxidase, urease, and arginine dihydrolase, tests for nitrate reduction, the production of indole, the utilization of citrate, and acid and gas production from glucose. Different carbon sources (d-Lactose, d-Glucose, d-Fructose, d-Maltose, Mannose, Xylose, d-Rhamnose, d-Mannitol, and d-Sorbitol) were used to evaluate carbon utilization. Except for the gelatinase activity test (which was performed at 20 °C), all of the tests were performed at 28 °C in the appropriate medium and were conducted according to standard methods [69].

## 4. Conclusions

This study demonstrates that the larvae of *H. parallela* harbor a dense and diverse community of cellulolytic bacteria in their hindgut and that the bacteria in the hindgut have an important role in the degradation of the roots and other organic matter consumed by scarab larvae. The 21 species of cellulolytic bacteria represent 17 genera, with the cellulolytic activity varying among the different strains, indicating that cellulolytic bacteria possess a significant amount of genetic diversity. Moreover, many bacterial species were reported to be cellulolytic for the first time in this study, which demonstrates that the scarab gut has a great potential to be a source of novel cellulolytic microorganisms and enzymes useful for future biofuel production.

## Figures and Tables

**Table 1:** The group identities and number of isolates.

Group	Representative strains	Phylum/class	Identities of isolates	Numbers of Strains	

				Medium II	Medium III
1	H16	Firmicutes	*Bacillus licheniformis*	6	3
2	H212	Bacteroidetes	*Dyadobacter fermentans*	2	0
3	H59		*Siphonobacter aquaeclarae*	0	1
4	H99	Actinobacteria	*Cellulosimicrobium funkei*	16	12
5	H97		*Microbacterium oxydans*	1	1
6	H63		*Microbacterium binotii*	5	4
7	H1		*Microbacterium pumilum*	2	9
8	H122	α-Proteobacteria	*Paracoccus sulfuroxidans*	3	0
9	H108		*Ochrobactrum lupini*	1	0
10	H191		*Ochrobactrum cytisi*	10	12
11	H70		*Ochrobactrum haematophilum*	2	0
12	H87		*Rhizobium radiobacter*	18	6
13	H6		*Kaistia adipata*	0	2
14	H162		*Devosia riboflavina*	0	1
15	H37		*Labrys neptuniae*	1	1
16	H75		*Ensifer adhaerens*	2	0
17	H173	β-Proteobacteria	*Variovorax paradoxus*	1	0
18	H19		*Shinella zoogloeoides*	0	1
19	H143	γ-Proteobacteria	*Citrobacter freundii*	5	0
20	H45		*Pseudomonas nitroreducens*	40	25
21	H72		*Stenotrophomonas maltophilia*	11	3

**Table 2:** Physiological and biochemical characteristic of isolated strains.

Characteristic	Representative Strains																				

	H16	H212	H59	H97	H99	H63	H1	H122	H108	H191	H87	H6	H162	H37	H70	H75	H173	H19	H143	H45	H72
Gram strain	+	−	−	+	+	+	+	−	−	−	−	−	−	−	−	−	−	−	−	−	−
Motility	+	−	−	+	+	−	−	−	−	+	+	−	+	−	−	+	+	+	+	+	+
Catalase	+	+	+	+	+	+	+	+	+	+	+	+	+	−	−	+	+	+	+	+	+
Oxidase	+	+	−	−	+	−	−	+	+	+	+	+	+	−	+	+	+	+	−	−	−
MR test	+	−	+	−	+	+	−	−	+	+	−	−	−	−	−	−	+	−	+	−	−
V-P test	+	−	−	−	−	−	−	−	−	+	−	−	−	−	−	−	+	−	+	−	−
Indole test	−	−	−	−	+	−	−	−	−	+	+	−	−	−	+	−	−	−	−	−	−
Nitrate reduction	+	−	−	−	−	−	+	+	+	+	+	−	+	+	+	+	+	−	+	+	−
Urease	−	−	−	−	+	−	−	+	+	−	+	+	+	+	−	−	+	−	+	+	−
**Hydrolysis of**																					
Starch	+	−	+	−	+	+	−	−	+	−	−	−	−	−	−	−	−	−	−	−	+
Gelatin	+	−	+	+	+	+	−	−	−	−	−	+	−	−	−	−	−	+	−	−	+
Acid produced from glucose	+	+	+	+	+	+	−	−	+	−	+	−	−	−	−	+	−	+	+	+	+
Gas produced from glucose	+	+	−	−	−	−	−	−	−	−	−	−	−	+	−	−	−	−	+	−	+
Arginine dihydrolase	+	−	−	−	+	−	+	−	−	−	+	−	−	−	−	+	−	−	−	−	−
**Assimilation of**																					
Citrate	+	+	+	+	+	−	+	+	−	+	−	−	−	−	+	+	+	−	+	+	+
Fructose	+	+	+	+	+	+	−	−	−	+	+	+	+	+	+	+	+	+	−	+	+
Glucose	+	+	+	+	+	+	+	+	+	+	+	+	+	+	+	+	+	+	+	+	+
Lactose	−	+	+	+	+	+	−	−	+	+	+	+	+	−	+	+	+	+	+	+	−
Maltose	+	+	+	+	+	+	+	+	+	+	+	+	+	−	+	+	+	+	−	−	+
Mannose	+	+	+	+	+	+	+	−	+	+	+	+	+	+	+	+	+	+	−	−	+
Mannitol	+	+	−	+	+	+	+	−	+	+	+	+	+	+	+	+	+	+	+	+	−
Rhamnose	+	+	+	+	+	+	−	−	+	+	+	+	+	+	+	+	+	+	+	−	+
Xylose	+	+	+	+	+	+	−	−	+	+	+	+	+	−	+	+	+	+	−	+	−
Sorbitol	−	+	−	+	+	+	−	−	−	+	−	+	+	+	+	+	+	+	−	−	−

Symbols: + positive; − negative.
